# Incorrect use and presentation of the RADNER Reading Charts: comment on measurement of reading speed with standardized texts: a comparison of single sentences and paragraphs, by Altpeter E, Marx T, Nguyen N, Naumann A, Trauzettel-Klosinski S

**DOI:** 10.1007/s00417-015-3183-z

**Published:** 2015-10-13

**Authors:** Wolfgang Radner, Kristel Maaijwee, Marc D. de Smet, Thomas Benesch

**Affiliations:** Austrian Academy of Ophthalmology, Mollgasse 11, 1180 Vienna, Austria; Department of Ophthalmology, HagaZiekenhuis, Leyweg 275, 2545 CH The Hague, The Netherlands; MIOS, Lausanne, Switzerland; Baumgasse 16, 1030 Vienna, Austria

Dear Editor,

We have read with interest the article of Alpeter et al. We appreciate the fact that they compared their reading speed determination with sentences derived from the RADNER Reading Charts [1, 2]. However, our reading charts were never designed to determine reading speed based on just a single sentence; this limited use is methodologically incorrect, and the comparison is therefore not appropriate. Further remarks regarding their analysis are provided below.

**Fig. 1:**
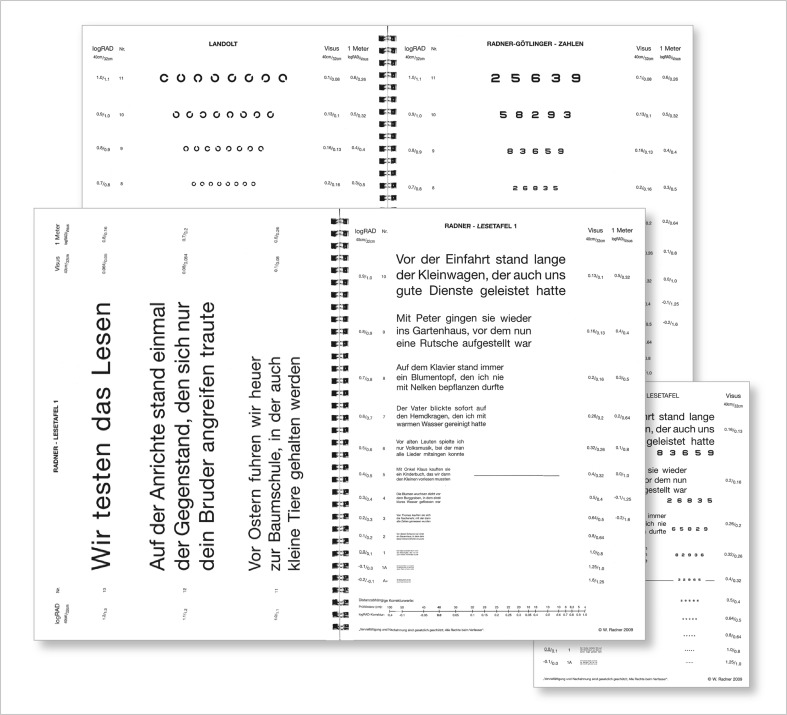
The RADNER Reading Charts, currently available in German, English, French, Spanish, Portuguese, Dutch, Italian, Danish, Swedish, Hungarian, and Turkish

The RADNER Reading Charts were designed for clinical and research use, providing a number of different reading parameters from a single examination in patients with normal to low vision [e.g., 1–3]. These reading charts offer standardized “sentence optotypes” [e.g., 1] that logarithmically progress in print size (Fig. 1). The sentence optotypes, developed in 11 different languages, have been standardized by reliability and validity analyses in 1,253 individuals involving over 42,000 measurements. The reliability of the RADNER Reading Charts has been analyzed by a test–retest protocol (interval, 3–4 weeks), interchart reliability analysis, and variant component analysis (randomized, orthogonal Latin square design) [2, 3].

The sentences optotypes have consistently given correlations of *r* ≈ 0.9 with long paragraphs. Analyzing different reading parameters (such as reading acuity, mean and maximum reading speed, and critical print size, among others, has proven advantageous in evaluating specific alterations of reading performance in different eye diseases [e.g., 4–8].

The RADNER Reading Charts have been used incorrectly in the study of Alpeter et al. All RADNER Reading Charts come with clear instructions included in the booklet, informing an examiner how to use them and how to reduce inaccuracies. The unusually high number of outliers among the 30 patients (who read only three sentences each) in the paper of Alpeter et al. suggests that the readers had not been instructed correctly. It is inappropriate that the participants recruited were friends and relatives of co-workers. In addition, there was no randomization.

There is incongruence between the results and our data. Using the data from a recent study (Radner et al., publication submitted) we calculated the correlations among seven sentence optotypes (font: Times Roman, 12 Pt), as measured in 60 normally sighted persons (Table 1). Our results offer a higher correlation (*r* = 0.775 to *r* = 0.909; *p* < 0.001; SPSS for Windows 21.0) than that of Alpeter et al., yet our examiner was a student well-instructed and experienced in the procedure.

**Table 1:** Correlations among seven sentence optotypes (S), presented randomized to 60 individuals

Pairs compared	*N*	Correlation
Pair 1 S1 & S2	60	0.855
Pair 2: S1 & S3	60	0.853
Pair 3: S1 & S4	60	0.878
Pair 4: S1 & S5	60	0.812
Pair 5: S1 & S6	60	0.829
Pair 6: S1 & S7	60	0.775
Pair 7: S2 & S3	60	0.893
Pair 8: S2 & S4	60	0.909
Pair 9: S2 & S5	60	0.840
Pair 10: S2 & S6	60	0.847
Pair 11: S2 & S7	60	0.829
Pair 12: S3 & S4	60	0.901
Pair 13: S3 & S5	60	0.867
Pair 14: S3 & S6	60	0.862
Pair 15: S3 & S7	60	0.777
Pair 16: S4 & S5	60	0.867
Pair 17: S4 & S6	60	0.864
Pair 18: 4 & S7	60	0.785
Pair 19: S5 & S6	60	0.890
Pair 20: S5 & S7	60	0.811
Pair 21: S6 & S7	60	0.848

Some of possible sources of methodical inaccuracies that can appear are (to name just a few):

1. For accurate measurements of reading speed, we recommend to (a) look at the pre-phonetic lip strain [1], (b) use digital recordings or video-recordings for the measurements [3], or (c) use our automated computer program for reading acuity and speed [9].
2. It is not possible to compare a reading test with long paragraphs meant for testing reading speed alone to a reading chart with a logarithmic progression of print sizes. In particular, one should not take out of context only three sentence optotypes out of 38, calculate a correlation between the sentences, and compare this with the correlations between long paragraphs (selection bias). There are several methods to test the reliability of a method, such as Cronbach’s alpha [1], and there are many highly standardized reading tests with long paragraphs available to show the validity of the IReST (e.g., in psychology).
3. We recommend increasing the number of presented text passages and calculating a mean reading speed ±SD in order to increase the reliability of the procedure. This was the principal thought when we developed the RADNER Reading Charts. We also recommend presenting sets of two or more paragraphs of the IReST, particularly for comparative reading speed analyses, because Brussee et al. found significant differences (*p* < 0.05) between paragraphs of the IReST [10], and we have found significant differences (*p* < 0.05) in reading speed between long paragraphs that have been developed with equivalent sentence construction, number of words (111), number of characters (660), number of syllables (179), and positions of words with the same number of syllables (Radner et al., publication submitted; the paragraphs were approved by a linguist; 60 persons were studied).

In summary, we do not agree with the conclusions of Altpeter et al., since their methodology leads to incongruent measurements and thus the risk of aberrant interpretation. We would like to emphasize that previous studies from different groups have provided evidence of the high reliability and validity of the RADNER Reading Charts.
